# Medication Adherence Across Diseases Using the 8-Item Morisky Medication Adherence Scale (MMAS-8): A Systematic Review With Narrative Synthesis

**DOI:** 10.7759/cureus.112863

**Published:** 2026-07-17

**Authors:** Esteban Zavaleta-Monestel, Jeaustin Mora-Jiménez, Sebastián Arguedas-Chacón, Luis Carlos Monge Bogantes, Andy Hernandez Vasquez, Philip Magueflor Morisky

**Affiliations:** 1 Department of Research, Hospital Clínica Bíblica, San José, CRI; 2 Department of Pharmacy, Hospital Clínica Bíblica, San José, CRI; 3 College of Business, University of La Verne, La Verne, USA

**Keywords:** chronic disease managment, medication adherence strategies, morisky medication adherence scale-8, noncommunicable diseases, pharmacist intervention

## Abstract

Medication adherence is a key determinant of clinical outcomes in patients with noncommunicable chronic diseases, where long-term pharmacotherapy, continuous follow-up, and patient engagement are often required. This systematic review evaluated the use of the 8-item Morisky Medication Adherence Scale (MMAS-8) to assess medication adherence in adults with noncommunicable chronic diseases and summarized the adherence levels, associated factors, and methodological characteristics reported across clinical settings. The review was conducted according to the Preferred Reporting Items for Systematic Reviews and Meta-Analyses (PRISMA) 2020 statement. Searches were performed in PubMed, ScienceDirect, and Google Scholar for studies published between January 2020 and September 2025. Eligible studies included primary research articles in English or Spanish that used the MMAS-8 as the main tool for assessing medication adherence in adults. A total of 989 records were identified. After removal of 71 duplicates, 918 records were screened by title and abstract, and 27 full-text articles were assessed for eligibility. Finally, 19 studies met the inclusion criteria and were included in the qualitative synthesis. The included studies evaluated conditions such as hypertension, diabetes mellitus, cardiovascular disease, breast cancer, psoriasis, hypothyroidism, depression, multiple sclerosis, chronic pain, and multimorbidity. Overall, low or medium adherence was frequently reported, although adherence levels varied according to disease, care setting, population characteristics, and method of scale administration. Factors associated with adherence included sociodemographic characteristics, family support, health literacy, adverse effects, treatment satisfaction, quality of life, and pharmacist-led interventions. The MMAS-8 appears to be a practical tool for identifying potential medication adherence problems in chronic disease populations; however, its self-reported nature, methodological heterogeneity, and variability in administration limit direct comparability across studies. Future research should standardize the MMAS-8 application and combine it with objective adherence measures.

## Introduction and background

Therapeutic adherence is defined as the extent to which a patient’s behavior, including correct medication use, attendance at follow-up appointments, and compliance with health-related recommendations, aligns with the instructions agreed upon with healthcare professionals [1]. In patients with noncommunicable chronic diseases, medication adherence is an essential component of treatment, as it directly influences clinical control, disease progression, mortality, and healthcare-related costs [2]. In 2021, noncommunicable diseases accounted for 43.8 million deaths worldwide, representing 64.5% of all deaths, with an age-standardized mortality rate of 554.63 per 100,000 population [3].

Noncommunicable chronic diseases, including cardiovascular diseases, cancer, chronic respiratory diseases, and diabetes, often require long-term pharmacological treatment, continuous clinical follow-up, and lifestyle modifications [4]. However, suboptimal adherence remains a frequent barrier to achieving adequate therapeutic outcomes, particularly in settings with economic constraints, limited access to healthcare services, or high medication costs [5]. For this reason, accurate assessment of adherence is essential to identify therapeutic failures, guide clinical interventions, explain potentially avoidable hospitalizations, and estimate the economic impact of inadequate adherence [6].

The selection of an adherence measurement instrument should consider its conceptual structure, psychometric properties, clinical applicability, and performance across different populations [2]. In this context, the 8-item Morisky Medication Adherence Scale (MMAS-8) has been widely used as a self-report questionnaire to assess medication-taking behavior and classify therapeutic adherence into high, medium, or low levels [7-10]. The scale includes items related to forgetfulness, carelessness, treatment interruption when feeling better or worse, difficulties remembering medication, and general adherence behavior. However, its interpretation requires caution because MMAS-8 scores represent an estimate of adherence behavior rather than a direct measure of medication intake. In addition, results may be influenced by recall bias, social desirability, cultural differences, health literacy level, mode of administration, validated translation, and variations in the cut-off points used across studies.

Recent literature shows wide variability in adherence levels measured with the MMAS-8 among patients with noncommunicable chronic diseases, as well as in the factors associated with higher or lower adherence. These factors include sociodemographic characteristics, family support, adverse effects, treatment satisfaction, quality of life, health literacy, and pharmacist involvement in the care process. However, the available evidence is dispersed across different diseases, regions, and methodological designs, which makes an integrated interpretation of the findings difficult.

In this context, the objective of this systematic review is to analyze the scientific evidence on therapeutic adherence levels measured using the MMAS-8 in patients with noncommunicable chronic diseases, as well as to identify the main associated factors and the methodological characteristics of its application across different clinical settings.

## Review

Methodology

This systematic review was developed and reported according to the Preferred Reporting Items for Systematic Reviews and Meta-Analyses (PRISMA) 2020 statement [11].

PROSPERO Registration

The review protocol was registered in the International Prospective Register of Systematic Reviews (PROSPERO) database under the registration number CRD420261285523. The research question, eligibility criteria, and outcomes of interest were predefined. No substantial modifications to the protocol were made during the development of the review.

Search Strategy

A structured literature search was conducted in PubMed, ScienceDirect, and Google Scholar. The search included studies published between January 2020 and September 2025. This period was selected to provide a contemporary synthesis of MMAS-8 use in chronic disease populations, particularly after recent changes in chronic disease care delivery, digital health tools, telemedicine, and pharmacist-led adherence interventions. Foundational studies on the MMAS-8 were used for conceptual background but were not part of the eligibility window for the qualitative synthesis. The strategy combined controlled vocabulary, when available, and free-text terms related to medication adherence, chronic diseases, and the MMAS-8 scale. In PubMed, MeSH terms were combined with keywords, whereas in ScienceDirect and Google Scholar, free-text terms were adapted to the characteristics of each platform.

The search terms included combinations of the following: “Medication Adherence,” “adherence,” “compliance,” “MMAS-8,” “Morisky Medication Adherence Scale,” “Morisky scale,” “Chronic Disease,” “chronic illness,” and “noncommunicable disease.”

All records retrieved from PubMed, ScienceDirect, and Google Scholar were compiled in a standardized spreadsheet. Duplicate records were identified using the digital object identifier (DOI) when available. When DOI was not available, records were manually compared using title, first author, journal, and year of publication. Google Scholar records were manually cross-checked against PubMed and ScienceDirect records before title and abstract screening, and duplicate entries were removed before screening.

Eligibility Criteria

Eligibility criteria were defined according to the research question. Primary studies published in English or Spanish that assessed medication adherence using the MMAS-8 scale in adult patients with noncommunicable chronic diseases were included. Observational, cross-sectional, analytical, validation, and randomized clinical trial designs were eligible, provided that they reported quantifiable data related to therapeutic adherence.

Only studies involving adult populations, defined as participants aged 18 years or older, and with a minimum sample size of 50 subjects, were included. This threshold was established to favor the inclusion of studies with greater stability in their estimates and to reduce variability associated with small samples.

Systematic reviews, meta-analyses, narrative reviews, editorials, letters to the editor, protocols without results, pediatric studies, studies with sample sizes below 50 participants, articles without full-text availability, studies that did not use the MMAS-8 as the main measurement tool, and studies focused on conditions outside the scope of this review were excluded.

Study Selection

Two reviewers independently assessed the titles and abstracts of all identified records. Potentially eligible articles were subsequently reviewed in full text. Discrepancies between reviewers were resolved through discussion and consensus. When agreement could not be reached, a third reviewer was consulted.

Data Extraction

Data extraction was performed independently by two reviewers using a predesigned extraction sheet. The following variables were collected: author, year of publication, country, study design, sample size, population characteristics, disease evaluated, healthcare setting, mode of MMAS-8 administration, cut-off points used, adherence levels reported, associated factors, presence or absence of an intervention, and main findings.

When studies reported interventions aimed at improving adherence, additional information was extracted regarding the type of intervention, duration of follow-up, professionals involved, and observed changes in adherence levels.

Risk of Bias and Methodological Quality Assessment

Two reviewers independently assessed the methodological quality of the included studies. Randomized clinical trials were evaluated using the Risk of Bias 2 (RoB 2) tool. Observational, cross-sectional, and non-randomized studies were assessed using the Joanna Briggs Institute (JBI) critical appraisal checklists [12].

No study was excluded solely on the basis of methodological quality assessment. Risk-of-bias findings were used as an interpretive element to contextualize the strength of the evidence.

Validation studies of the MMAS-8 were included when they reported data on the application of the scale in clinical populations. However, because psychometric properties were not the primary objective of this review, their findings were interpreted with caution.

Data Synthesis

A meta-analysis was not performed because of clinical, methodological, and measurement heterogeneity among the included studies. This heterogeneity was related to differences in the diseases evaluated, study designs, healthcare settings, modes of MMAS-8 administration, cut-off points used, and outcomes reported.

Therefore, a structured narrative synthesis of the evidence was conducted. First, studies were grouped according to disease or population group. Second, adherence levels were summarized according to the categories reported by each study. Third, associated factors were organized into sociodemographic, clinical, treatment-related, behavioral, and health-system-related domains. Finally, studies reporting interventions were described separately because of differences in intervention design, duration, setting, and outcome reporting.

Because adherence categories, cut-off points, and administration modes differed across studies, results were interpreted comparatively rather than pooled quantitatively. The synthesis was organized according to the characteristics of the included studies, reported adherence levels, factors associated with adherence, interventions described, and methodological limitations related to the use of the MMAS-8.

Results

Study Selection

The structured search in PubMed, ScienceDirect, and Google Scholar identified a total of 989 records. After the removal of 71 duplicates, 918 records were screened by title and abstract. Of these, 891 records were excluded because they did not meet the eligibility criteria or were not aligned with the objective of the review.

**Figure 1:**
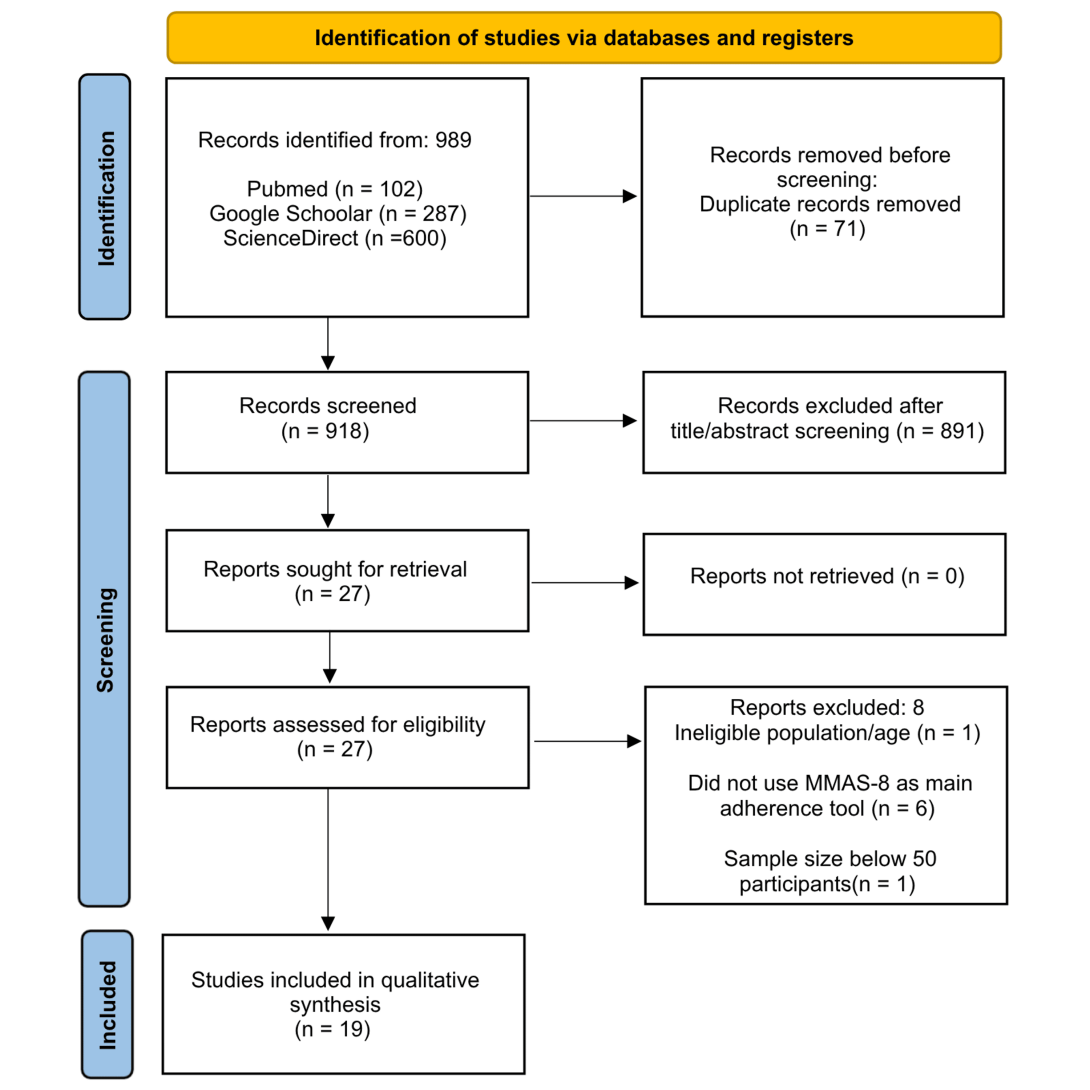
PRISMA 2020 flow diagram of the study selection process. Flow diagram summarizing the identification, duplicate removal, title and abstract screening, full-text assessment, and final inclusion of studies that evaluated therapeutic adherence using the MMAS-8 in patients with noncommunicable chronic diseases. PRISMA: Preferred Reporting Items for Systematic Reviews and Meta-Analyses; MMAS-8: 8-Item Morisky Medication Adherence Scale

A total of 27 full-text articles were sought for retrieval and assessed for eligibility. No reports were unavailable for retrieval. Eight articles were excluded at this stage: six did not use the MMAS-8 as the main medication adherence assessment tool, one included an ineligible population or age group, and one had a sample size below 50 participants. Finally, 19 studies met all eligibility criteria and were included in the qualitative synthesis. The identification, screening, eligibility assessment, and inclusion process are summarized in Figure 1.

Characteristics of the Included Studies

The general characteristics of the 19 included studies are summarized in Table 1. The studies were exclusively primary research articles that used the MMAS-8 to assess medication adherence in adult patients with noncommunicable chronic diseases. The study designs included cross-sectional studies, observational studies, validation studies, and randomized clinical trials.

**Table 1:** Characteristics of the included studies that used the MMAS-8 to assess medication adherence in noncommunicable chronic diseases. MMAS-8: 8-Item Morisky Medication Adherence Scale; MUR: Medicines Use Review; RCT: randomized controlled trial

Author	Country	Disease	Design	n	Care setting	MMAS-8 administration	Cut-off points	Intervention	Main finding
Satish et al. [7]	India (Karnataka)	Hypertension	Cross-sectional	150	Clinic	Self-administered	Standard	No	Knowledge and practices are associated with adherence
Ghosh et al. [8]	India	Diabetes	Cross-sectional	178	Clinic	Interview	Standard	No	Environmental barriers affect adherence
Zhang et al. [9]	China	Chronic pain	Validation	113	Clinic	Self-administered	Modified	No	MMAS-8 validated in a specific population
Santi et al. [13]	India	Depression	Clinical trial	71	Hospital	Interview	Standard	Yes	Treatment influences adherence and quality of life
ElBaghdady et al. [14]	Egypt	Breast cancer	Cross-sectional	300	Hospital	Interview	Standard	No	Low adherence to hormonal therapy
Avazeh et al. [15]	Iran	Psoriasis	Cross-sectional	575	Clinic	Electronic	Standard	No	Low adherence is associated with health literacy
Nabergoj et al. [16]	Slovenia	Chronic diseases	RCT	170	Community pharmacy	Electronic	Standard	Yes	MUR improves adherence
Garcia et al. [17]	Mexico	Hypertension	Cross-sectional	127	Clinic	Electronic	Standard	No	Family support improves adherence
Rosa et al. [18]	Brazil	Hypertension	Cross-sectional	302	Community	Interview	Standard	No	Sociodemographic factors have an influence
Rabeea et al. [19]	Iraq	Breast cancer	RCT	75	Hospital	Interview	Standard	Yes	Pharmaceutical intervention improves adherence
Alhemedi et al. [20]	Jordan	Hypothyroidism	Cross-sectional	296	Clinic	Self-administered	Standard	No	Depression is associated with lower adherence
Alalaqi et al. [21]	Iraq	Cardiovascular	Mixed	303	Hospital	Self-administered	Standard	No	Differences between measurement methods
Santi et al. [22]	China	Depression	RCT	134	Hospital	Interview	Standard	Yes	Improved adherence with treatment
Munsour et al. [23]	Qatar	Type 2 diabetes	RCT	140	Clinic	Interview	Standard	Yes	Education improves adherence
Khedr et al. [24]	Egypt	Multiple sclerosis	Cross-sectional	97	Hospital	Self-administered	Standard	No	Satisfaction predicts adherence
Khaiser et al. [25]	India	Chronic diseases	Cross-sectional	310	Hospital	Self-administered	Standard	No	Relationship with quality of life
Gavrilova et al. [26]	Latvia	Hypertension	Cross-sectional	187	Community	Interview	Standard	No	Factors influence non-adherence
Xia et al. [27]	China	Chronic diseases	Observational	424	Community pharmacy	Self-administered	Standard	Yes	Pharmaceutical intervention improves adherence
Allela et al. [28]	Iraq	Type 2 diabetes	Cross-sectional	307	Clinic	Self-administered	Standard	No	Validation and adherence levels

The diseases evaluated included arterial hypertension, diabetes mellitus, cardiovascular disease, breast cancer, psoriasis, hypothyroidism, depression, multiple sclerosis, chronic pain, and populations with multiple chronic diseases. Healthcare settings were heterogeneous and included hospital care, outpatient care, specialized clinics, community pharmacies, and community-based settings.

The MMAS-8 was administered using different modalities, including interviews, self-administration, and electronic formats. Most studies used the standard cut-off points to classify adherence as high, medium, or low; however, some studies used adapted or modified versions of the scale. This methodological variability limited direct comparability across studies.

Levels of Therapeutic Adherence Measured With the MMAS-8

The adherence levels reported varied widely across the included studies. In general, most investigations described a predominance of low or medium adherence, although the magnitude of these findings depended on the disease evaluated, the care setting, the mode of scale administration, and the characteristics of the population [7-9,13-28].

In studies conducted in patients with arterial hypertension, adherence tended to fall between low and medium levels, with reported associations with sociofamilial support, age, educational level, and characteristics of access to care [7,17,18]. In patients with diabetes mellitus, low adherence was frequent and was related to self-care barriers, environmental factors, and limited understanding of treatment [8,28]. In breast cancer, adherence levels were variable, especially in patients receiving oral hormonal therapy, where adverse effects and sociodemographic factors influenced treatment continuity [14,19]. In psoriasis, a high proportion of low adherence was reported, mainly associated with side effects and health literacy [15]. The adherence trends reported by condition are summarized in Table 2.

**Table 2:** Adherence levels reported by condition or population group.

Condition or population group	Number of studies	General adherence pattern	Main associated factors
Hypertension [7,17,18,26]	4	Low to medium	Sociofamilial support, age, income, access to care, and knowledge
Diabetes mellitus [8,23,28]	3	Mostly low	Self-care barriers, environmental barriers, and patient education
Breast cancer [14,19]	2	Variable	Adverse effects, sociodemographic factors, and pharmacist follow-up
Psoriasis [15]	1	Low	Health literacy and side effects
Other chronic diseases or populations [9,13,16,20–22,24,25,27]	9	Variable	Quality of life, treatment satisfaction, polypharmacy, intervention type

Factors Associated With Medication Adherence

The factors associated with therapeutic adherence were multifactorial and varied according to the disease, clinical context, and individual patient characteristics. Among the sociodemographic factors reported were age, educational level, marital status, employment situation, income level, and family cohabitation.

In patients with breast cancer, educational level, marital status, and employment status were associated with variations in adherence levels [14,19]. Similarly, in patients with arterial hypertension, factors such as age, income level, and living with family members were related to higher levels of medication adherence [7,17,18]. These findings suggest that social support and socioeconomic conditions can have a relevant influence on therapeutic compliance.

Other clinical and treatment-related factors were also relevant. Adverse effects were associated with lower adherence in conditions such as breast cancer and psoriasis, while treatment satisfaction and perceived clinical benefit were linked to better adherence levels in chronic diseases requiring prolonged treatment. Health literacy and the patient's knowledge of their disease and medication were also important determinants, particularly in studies that evaluated educational or pharmaceutical interventions.

Taken together, the included studies show that medication adherence does not depend on a single factor, but on the interaction of clinical, social, economic, behavioral, and health-system-related variables.

Interventions Aimed at Improving Adherence

Some included studies evaluated interventions aimed at improving therapeutic adherence, mainly through patient education, pharmaceutical follow-up, medication use review, and clinical accompaniment. Some of these studies reported improvements in MMAS-8 adherence scores after pharmacist-led or educational interventions. However, these findings should be interpreted as exploratory because the studies differed in intervention design, intensity, follow-up duration, clinical setting, and outcome reporting. Therefore, although these interventions appear promising, the current evidence does not allow firm conclusions regarding their comparative effectiveness.

Table 3 shows that pharmaceutical interventions were especially relevant in studies conducted in community pharmacies, hospitals, and outpatient clinics. These strategies made it possible to identify barriers related to treatment knowledge, resolve doubts about medication, reinforce the importance of therapeutic compliance, and promote greater patient participation in the management of their disease.

**Table 3:** Interventions based on MMAS-8 results and their impact on therapeutic adherence. MMAS-8: 8-Item Morisky Medication Adherence Scale

Study	Results
Nabergoj et al. [16]	The implementation of Medication Use Review (MUR) showed an improvement in adherence among patients
Rabeea et al. [19]	Pharmacist intervention through education and follow-up of patients shows a considerable impact on optimizing adherence
Munsour et al. [23]	The provision of medication information to patients improved adherence
Gavrilova et al. [26]	The implementation of an interview with the pharmacist was shown to improve adherence in patients.
Xia et al. [27]	Patient education on treatment and disease showed an improvement in adherence

Risk-of-Bias Assessment

**Figure 2:**
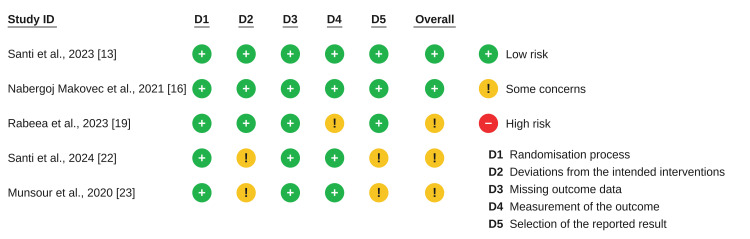
Risk-of-bias assessment of five included studies using the ROB-2 tool. [13,16,19,22,23]

Two reviewers independently assessed the risk of bias and methodological quality of the included studies. Randomized clinical trials were assessed using the RoB 2 tool. [29] . Figure 2 summarizes the results of this assessment. Some studies presented methodological concerns in domains related to deviations from the intervention, outcome measurement, or selection of the reported result. In particular, Santi et al. [22] and Munsour et al. [23] showed weaknesses in domains 2 and 5, while Rabeea et al. [19] presented concerns in domain 4.

**Figure 3:**
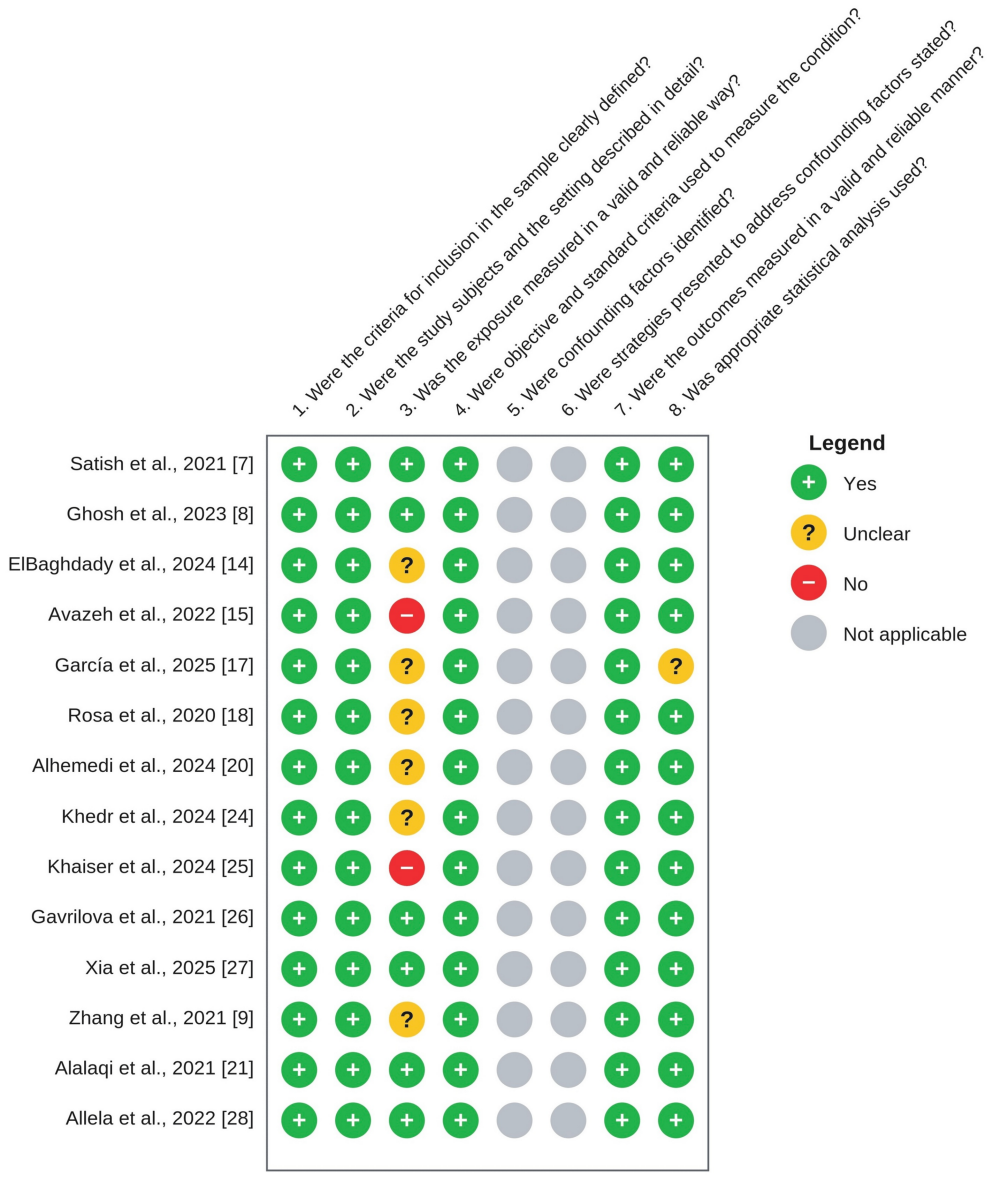
Methodological assessment of non-randomized studies using JBI tools. The figure shows the critical appraisal of the non-randomized studies included in the review, according to the Joanna Briggs Institute (JBI) checklists [12]. [7-9,14,15,17,18,20,21,24-28]

Non-randomized studies were assessed using the JBI checklists. Figure 3 presents the methodological assessment of 14 non-randomized studies, including cross-sectional, observational, and validation studies. In general, the studies clearly described the population and outcomes of interest, although limitations were identified related to control of confounding factors, justification of sample size, comparability between groups, and the cross-sectional nature of most designs.

Validation studies of the MMAS-8 were included only when they reported data on the application of the scale in adult chronic disease populations and provided information relevant to adherence assessment. These studies were not included to synthesize psychometric properties as a primary outcome. Instead, they were used to describe how the MMAS-8 has been applied in specific clinical populations. Therefore, their findings were interpreted cautiously and were not weighted in the same way as interventional or observational adherence studies.

Discussion

Clinical Applicability of the MMAS-8 in Chronic Diseases

The findings of this systematic review show that the MMAS-8 has been used in various clinical contexts to assess medication adherence in patients with chronic diseases. Its main usefulness lies in its ability to classify patients into high, medium, or low adherence levels through a brief and easy-to-apply tool. This characteristic is relevant in diseases that require prolonged treatment, continuous follow-up, and active patient participation in managing their condition [14].

However, the interpretation of results obtained with the MMAS-8 must be made with caution. Although the scale allows the identification of possible adherence problems, it does not directly measure actual medication intake [14]. Because it relies on self-report, its results may be influenced by recall bias, social desirability, level of health literacy, cultural differences, and variations in mode of administration [18]. For this reason, the MMAS-8 should be understood as a clinical support tool and not as an absolute measure of therapeutic behavior.

**Figure 4:**
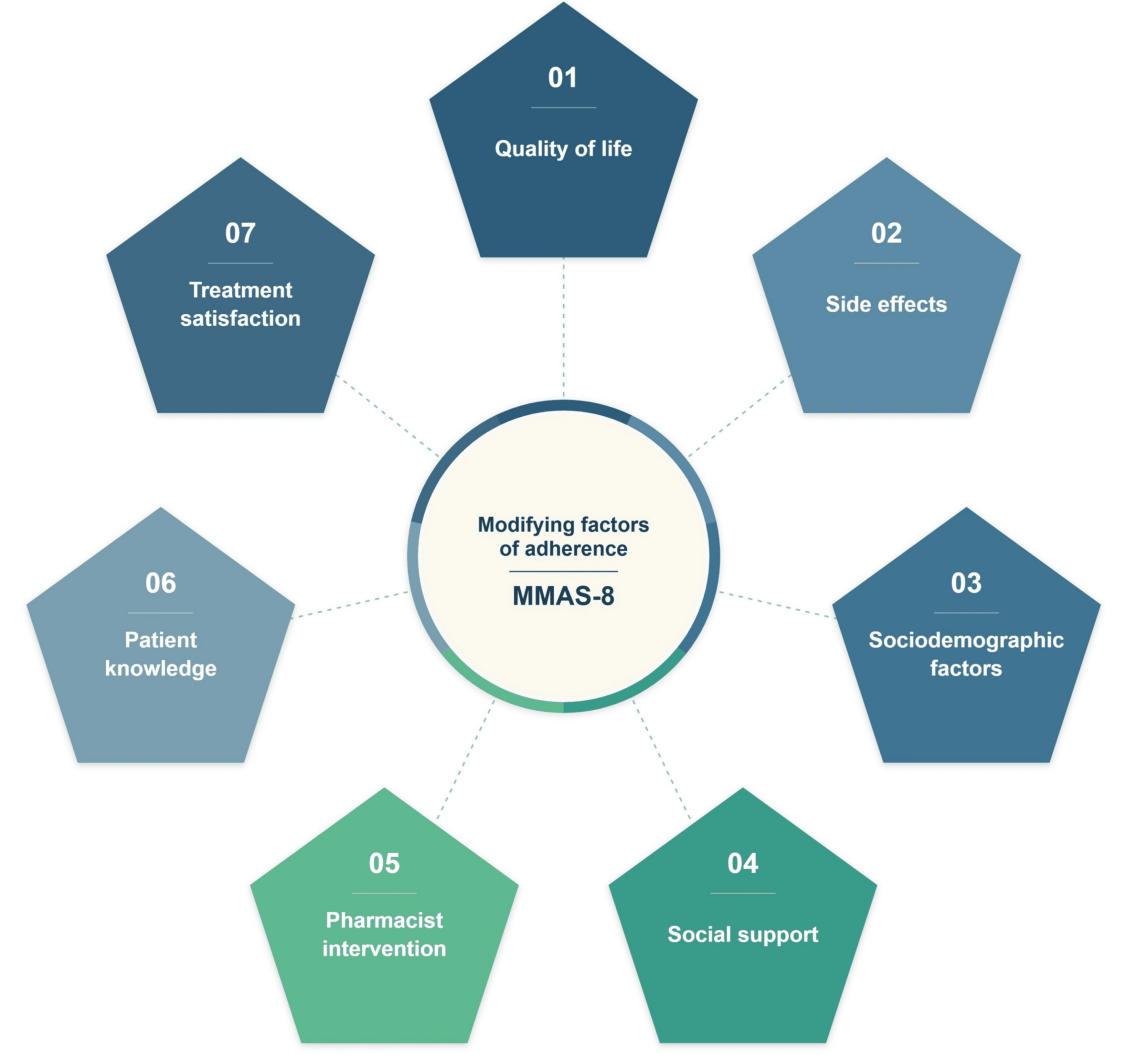
Conceptual model of the determinants of adherence identified in studies that used the 8-Item Morisky Medication Adherence Scale (MMAS-8). Schematic representation of the main factors associated with medication adherence, including quality of life, adverse effects, sociodemographic determinants, social support, treatment satisfaction, patient knowledge, and pharmaceutical interventions. This figure is an original creation by the authors, produced using Canva (Canva Pty Ltd, Surry Hills, NSW, Australia).

These factors and their link with adherence outcomes are summarized in Figure 4, which presents a conceptual model of the main variables that modulate medication-taking behavior in patients with chronic diseases.

Quality of Life, Adverse Effects, and Treatment Perception

Perceived quality of life was one of the relevant factors associated with therapeutic adherence. In chronic diseases, where treatment is usually maintained over prolonged periods, the perception of clinical improvement may favor therapeutic continuity. In patients with depressive disorders, adherence scores were related to quality of life and the perception of therapeutic benefit [22]. Similarly, in older adults, a relationship has been observed between medication adherence and quality of life, suggesting that perceived well-being may influence the patient's willingness to maintain treatment [25].

However, adverse effects can have the opposite effect. In patients with psoriasis, a high proportion of low adherence was reported, associated with factors such as health literacy and the presence of side effects [15]. In breast cancer, adherence to oral hormonal therapy was also influenced by treatment characteristics and by discontinuation of medication without prior consultation with healthcare staff [14]. These findings suggest that adherence assessment should be accompanied by an active exploration of tolerability, risk perception, and treatment-related barriers.

From a clinical perspective, adherence not only favors better therapeutic outcomes but may also contribute to disease control, reduction of complications, and a better quality of life [6]. Nevertheless, this relationship must be interpreted as bidirectional: better quality of life may favor adherence, but greater adherence can also contribute to better clinical outcomes.

Sociodemographic Factors and Social Support

Sociodemographic factors showed variable associations with medication adherence. In some studies, variables such as age, sex, marital status, educational level, employment situation, and income were related to differences in adherence levels [7,14,17-19]. However, these associations were not uniform across conditions, indicating that the impact of each factor depends on the clinical and social context of the population evaluated [21].

In patients with breast cancer, educational level, marital status, and employment status were associated with variations in adherence to oral hormonal therapy [14]. In arterial hypertension, sociofamilial support was linked to better adherence levels, highlighting the importance of family accompaniment in diseases that require continuous treatment [17]. Similarly, living with family members and having support networks may facilitate therapeutic compliance by promoting reminders, accompaniment to appointments, and understanding of the medication regimen [18].

On the other hand, certain populations showed particularly high rates of non-adherence, such as patients with diabetes and psoriasis [8]. These findings suggest that groups with complex treatments, persistent symptoms, or lower health literacy may require more intensive, individualized, and sustained interventions over time [15].

Pharmaceutical Interventions and Patient Knowledge

As summarized in Table 3, several included studies evaluated interventions aimed at improving therapeutic adherence, including medication use review, patient education, pharmaceutical follow-up, and clinical accompaniment strategies [16]. Taken together, these interventions were associated with improvements in adherence levels measured with the MMAS-8, although the magnitude and consistency of the effect varied across studies.

Medication use review allows the identification of pharmacotherapy-related problems, the resolution of patient doubts, and the application of corrective interventions aimed at improving treatment use. In the study by Nabergoj et al., medication use review was associated with improved adherence in patients with chronic diseases [16]. Similarly, Xia et al. reported that a pharmaceutical intervention in patients with chronic diseases was associated with better adherence outcomes [27].

Educational interventions also appear relevant. In patients with type 2 diabetes, the provision of individualized medication information was associated with improved adherence [23]. In breast cancer, a pharmaceutical intervention based on education and follow-up contributed to optimizing adherence to oral hormonal therapy [19]. In addition, in arterial hypertension, the pharmacist interview made it possible to identify gaps in treatment understanding, which underscores the need for repeated educational strategies adapted to the patient's level of comprehension [26].

However, these results must be interpreted with caution. Not all included studies evaluated interventions, and among those that did, there was heterogeneity in design, follow-up duration, intervention intensity, and care setting. Therefore, although the evidence suggests that pharmacist involvement may improve adherence, more robust studies are still needed to confirm the sustained effect of these interventions on relevant clinical outcomes.

Treatment Satisfaction

Treatment satisfaction was another relevant determinant of adherence. When patients perceive that medication improves symptoms, reduces disease progression, or facilitates clinical control, greater confidence in the therapy may develop and, therefore, greater willingness to maintain it. In patients with multiple sclerosis, satisfaction with disease-modifying therapy was identified as a predictor of adherence [24].

This finding is important because therapeutic satisfaction integrates clinical and subjective dimensions. It does not depend solely on pharmacological efficacy, but also on tolerability, ease of administration, patient expectations, and communication with the healthcare team. For this reason, adherence assessment using the MMAS-8 should be complemented by clinical questions aimed at understanding the patient's experience with their treatment.

Intentional and Unintentional Non-Adherence: Clinical and Measurement Implications

This section is presented as a conceptual extension rather than as a synthesized finding of the included studies. Something absent from most MMAS-8-based analyses is the distinction between intentional non-adherence (INA) and unintentional non-adherence (UNA). INA refers to deliberate decisions by the patient not to take medication as prescribed, which is often driven by adverse effects, lack of perceived benefit, or treatment-related beliefs. Whereas UNA reflects unintentional omissions attributable to forgetfulness, confusion, or difficulties managing the treatment regimen [4]. Patients who miss doses because of adverse effects or treatment skepticism require different clinical approaches than those whose non-adherence arises from cognitive or logistical constraints.

Several of the factors identified across these studies line up with this framework. Adverse effects and treatment dissatisfaction are well-documented adherence barriers in psoriasis, multiple sclerosis, and hypertension, and reflect INA-related mechanisms. Forgetfulness, polypharmacy burden, and cognitive impairment in older adults are UNA-related phenomena. However, none of the included studies employed a formal INA/UNA classification framework alongside the MMAS-8, which represents a gap in the conceptual application of the scale and limits the interpretability of non-adherence patterns identified across the reviewed literature [4].

Renna et al. recently provided a rigorous empirical characterization of INA/UNA phenotyping in a large cohort of 1,144 hypertensive patients from Argentina using a validated extension of the MMAS-8 (VATAHTA Study) [4]. Their analysis found that INA accounted for 38.5% of non-adherent patients, UNA for 33.6%, and mixed non-adherence, reflecting simultaneous INA and UNA features, for the remaining 27.9%. Applying k-means clustering to MMAS-8 item-level response profiles, the authors identified four adherence-complexity phenotypes with distinct clinical characteristics. Two phenotypes were independently associated with hypertension control after adjustment for confounders: Phenotype 2, characterized by predominant INA, showed an odds ratio of 0.42 (95% CI 0.29-0.61) for adequate blood pressure control, while Phenotype 4, reflecting a mixed adherence pattern, yielded an OR of 0.68 (95% CI 0.49-0.94). Causal mediation analysis further demonstrated that the indirect effect of INA on clinical outcomes, operating through treatment complexity, was statistically significant (β = 0.004, p < 0.001). Internal consistency of the MMAS-8 in this validation sample was Cronbach’s α = 0.78, supporting the psychometric robustness of the instrument when paired with behavioral subtyping.

These findings establish the clinical and methodological value of incorporating INA and UNA classifications into MMAS-8-based research. Moving beyond a single total score allows researchers and clinicians to determine whether non-adherence is driven by modifiable beliefs or by structural and cognitive constraints, a distinction with direct therapeutic relevance. Future studies applying the MMAS-8 in chronic disease populations should consider integrating INA/UNA frameworks to enhance the clinical actionability of adherence assessments and to strengthen the explanatory power of observational and interventional research.

Measurement Limitations of the MMAS-8

Although the MMAS-8 is a practical and widely used tool, it has limitations inherent to self-report instruments. Patients may overestimate their adherence, forget episodes of omission, or respond according to what they consider socially acceptable [15,19]. Furthermore, in patients with polypharmacy, the scale may not accurately identify which medication corresponds to the lack of adherence, which limits its usefulness when a drug-specific assessment is required [21].

This limitation is especially important in older adults, where polypharmacy, poor memory, confusion, or the presence of cognitive impairment may affect the accuracy of responses [30]. Likewise, the MMAS-8 does not always allow the identification of the specific reasons for non-adherence or the complete characterization of individual patient barriers [31].

Another relevant aspect is the linguistic and cultural adaptation of the scale. The use of translated versions requires rigorous validation processes to preserve the conceptual meaning of each item and ensure that the tool maintains its measurement properties in the population evaluated [32]. For these reasons, when possible, the MMAS-8 should be complemented with other assessment methods, such as clinical interviews, dispensing records, pill counts, electronic monitoring, or pharmacological measurements [21].

Implications for Clinical Practice and Future Research

The results of this review suggest that the MMAS-8 may be useful as an initial tool to identify patients with possible suboptimal adherence. Its application can guide educational interventions, pharmacotherapeutic follow-up, and individualized support strategies. However, the score obtained must be interpreted together with the clinical history, medication regimen, presence of comorbidities, adverse effects, and the patient's social conditions.

Future research should employ longitudinal designs and clinical trials with sufficient follow-up to evaluate sustained changes in adherence. It would also be advisable to report in a standardized manner the version of the MMAS-8 used, the language, the mode of administration, the cut-off points applied, and complementary adherence measures. This standardization would improve comparability between studies and strengthen the clinical interpretation of the results.

Limitations 

This review has several limitations. First, substantial clinical, methodological, and measurement heterogeneity among the included studies prevented the performance of a meta-analysis. Studies differed in diseases and populations evaluated, study designs, healthcare settings, MMAS-8 administration modes, cut-off points, and reported outcomes, which limited direct comparison and the possibility of obtaining a pooled quantitative estimate.

Second, most included studies were cross-sectional or observational; therefore, associations between adherence and factors such as sociodemographic characteristics, health literacy, adverse effects, treatment satisfaction, or social support should be interpreted as exploratory rather than causal.

Third, the search was restricted to studies published in English or Spanish with full-text availability, which may have introduced language and publication bias. In addition, Google Scholar was used as a complementary source, but its results may vary over time and according to platform algorithms, limiting reproducibility.

Finally, the 2020-2025 time frame was selected to provide a contemporary synthesis of MMAS-8 use in chronic disease populations, but it may have excluded earlier foundational studies or older applications of the scale. Similarly, the exclusion of studies with fewer than 50 participants may have omitted potentially relevant smaller studies.

Despite these limitations, this review provides an updated synthesis of the use of the MMAS-8 in patients with noncommunicable chronic diseases and identifies relevant patterns of application, associated factors, methodological limitations, and priority areas for future research.

## Conclusions

The evidence analyzed indicates that the MMAS-8 is a useful tool for evaluating and classifying therapeutic adherence in patients with chronic diseases, facilitating the identification of patterns of pharmacological non-compliance. However, its results must be interpreted with caution due to the limitations inherent to self-report instruments, including recall bias, social desirability, and difficulty in identifying drug-specific adherence problems in patients with polypharmacy.

The included studies show substantial heterogeneity in terms of populations, diseases evaluated, care settings, modes of administration, and cut-off points used, which limits direct comparability of the findings. Likewise, although some pharmaceutical and educational interventions showed promising improvements in adherence measured with the MMAS-8, this evidence remains exploratory and should be interpreted cautiously because of methodological heterogeneity among studies.

Future research should use longitudinal designs, standardized MMAS-8 reporting, culturally validated versions, and complementary objective adherence measures. Greater methodological consistency would allow a more reliable evaluation of therapeutic adherence and of the effect of interventions aimed at improving adherence in different chronic disease populations.
